# Chromosomal Characterization of *Tripidium arundinaceum* Revealed by Oligo-FISH

**DOI:** 10.3390/ijms22168539

**Published:** 2021-08-09

**Authors:** Fan Yu, Jin Chai, Xueting Li, Zehuai Yu, Ruiting Yang, Xueer Ding, Qiusong Wang, Jiayun Wu, Xiping Yang, Zuhu Deng

**Affiliations:** 1 National Engineering Research Center for Sugarcane, Fujian Agriculture and Forestry University, Fuzhou 350002, China; yufanky@163.com (F.Y.); CJ1152648@163.com (J.C.); lxt19910226@163.com (X.L.); Angelinating2021@163.com (R.Y.); 15294800931@163.com (X.D.); 13110898682@163.com (Q.W.); 2 Key Lab of Sugarcane Biology and Genetic Breeding, Ministry of Agriculture, Fujian Agriculture and Forestry University, Fuzhou 350002, China; 3 State Key Laboratory for Protection and Utilization of Subtropical Agro-bioresources, Guangxi University, Nanning 530004, China; ArHuay_Yu@163.com (Z.Y.); xipingyang@gxu.edu.cn (X.Y.); 4 Institute of Nanfan & Seed Industry, Guangdong Academy of Sciences, Guangzhou 510316, China

**Keywords:** *T.* *arundinaceum*, sugarcane, sorghum, maize chromosome painting probes, oligo-FISH, ribosomal DNA, chromosome identification, karyotype

## Abstract

Sugarcane is of important economic value for producing sugar and bioethanol. *Tripidium* *arundinaceum* (old name: *Erianthus* *arundinaceum*) is an intergeneric wild species of sugarcane that has desirable resistance traits for improving sugarcane varieties. However, the scarcity of chromosome markers has hindered the cytogenetic study of *T. arundinaceum*. Here we applied maize chromosome painting probes (MCPs) to identify chromosomes in sorghum and *T. arundinaceum* using a repeated fluorescence in situ hybridization (FISH) system. Sequential FISH revealed that these MCPs can be used as reliable chromosome markers for *T. arundinaceum*, even though *T. arundinaceum* has diverged from maize over 18 MYs (million years). Using these MCPs, we identified *T. arundinaceum* chromosomes based on their sequence similarity compared to sorghum and labeled them 1 through 10. Then, the karyotype of *T. arundinaceum* was established by multiple oligo-FISH. Furthermore, FISH results revealed that 5S rDNA and 35S rDNA are localized on chromosomes 5 and 6, respectively, in *T. arundinaceum*. Altogether, these results represent an essential step for further cytogenetic research of *T. arundinaceum* in sugarcane breeding.

## 1. Introduction

Sucrose is produced from two major crops, sugarcane (*Saccharum* spp.) and sugar beet (*Beta vulgaris*). Sugarcane, however, accounts for the vast majority of global sugar production and provides feedstocks for bio-energy production. For sugarcane breeding, interspecific hybridization is a powerful way to enhance resistance but also provides unexpected benefits in increasing yield and improving ratooning ability and adaptability [1]. *S. spontaneum* (2n = 40–128), which for more than a century has played a major role in sugarcane breeding, has been widely applied to improve the resistance of sugarcane cultivars. Simultaneously, *E. arundinaceum* (2n = 60, *x* = 10) is also used for sugarcane breeding due its high biomass productivity, superior ratooning ability, and exceptional adaptability to biotic and abiotic stresses [2]. Most recently, Lloyd Evans et al. placed *E. arundinaceum* to the *Tripidium* genus based on the whole chloroplast genome [3]. Thus, *E. arundinaceum* was referred to *T. arundinaceum* accordingly. Currently, many studies have reported cytogenetic research on *T. arundinaceum*, especially on the chromosome inheritance of the hybrids between sugarcane and *T. arundinaceum* [4,5,6]. However, basic cytogenetic information such as the karyotype, and the precise chromosome contribution of *T. arundinaceus* in interspecific hybridization, could not be addressed due to lack of effective cytogenetic markers to identify the individual chromosomes.

FISH is a powerful tool that has widely answered the cytogenetics issues, such as karyotype, chromosome recombination, genetic relationship of the related species, and chromosome transmission [7]. Successful and efficient FISH needs a reliable probe, such as genomic DNA (gDNA), repetitive sequences, bacterial artificial chromosome (BAC) clones, and oligonucleotides (oligos) [7,8,9]. Of these, oligos are a recent development and application of a probe that can be computationally identified according to sequencing genome data [7,10]. Oligo probes now are classified as repetitive oligonucleotide (repetitive DNA sequence), chromosome barcode (a specific chromosomal region), and chromosome painting (an entire chromosome region), and have been successfully applied in many plants, i.e., rice [11], wheat [12], citrus [13], maize [14], etc. Oligos designed from genome sequences based on low-copy sequences are better conserved than the repetitive DNA, which can be used for chromosome identification in related species that have diverged for several million years (MYs), or even more than 15 MYs [13,15,16].

In the present study, we tested maize chromosome painting probes (MCPs) in sorghum and *T. arundinaceum* using multiple rounds of FISH. Based on the oligo-FISH results, for the first time, we reported that MCPs can distinctly detect chromosomes 1–10 of *T. arundinaceum*. Then, the karyotype of *T. arundinaceum* was established according to the individual chromosome identification combining MCPs, 5S rDNA, and 35S rDNA probes. Altogether, these results will be useful for further understanding the chromosome inheritance of *T. arundinaceum* and improving the efficiency of sugarcane breeding.

## 2. Results

### 2.1. Sequence Alignment Analysis between MCP Sequences and Sorghum Genome

**Figure 1:**
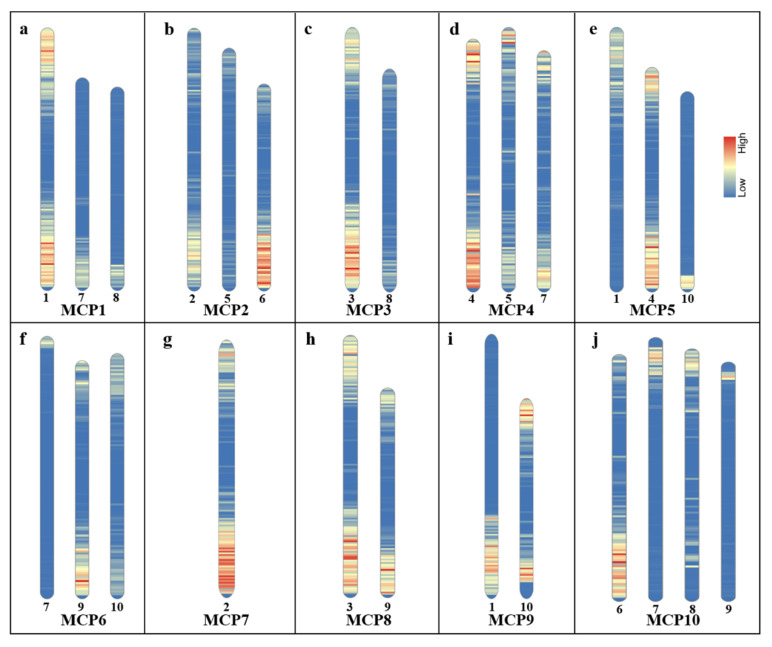
Maize CP sequences’ distribution on sorghum chromosomes. Arabic numerals indicate the chromosome of sorghum; (**a**–**j**) indicate the MCP1-10 sequence distributions on sorghum chromosomes in each 500 kb window, respectively.

We aimed to align 10 MCP sequences to 10 pseudomolecules of sorghum. Then, the sequence comparison was performed between MCP sequences and sorghum genome (see Methods section). The results showed that the number of oligos that align to the sorghum genome ranged from 277 to 27,169 (Table 1), which implied that these MCPs will produce various signals on the chromosomes of sorghum. To further understand the distribution of MCP sequences, we selected the MCP sequences that have the potential to produce obvious signals (the number of oligos > 1000) for displaying their locations on ten sorghum chromosomes (Figure 1); for example, MCP1 sequences aligned with sorghum chromosomes 1, 7, and 8 (Figure 1a and Figure S1). This implies that the MCP1 probe may produce hybridization signals on these three sorghum chromosomes. Although the MCP sequences showed an uneven distribution in sorghum, the number of the aligned MCP oligos was up to 10,000 on half of the sorghum chromosomes (chromosomes 1, 2, 3, 4, and 9, Table 1), and even 27,169 (chromosome 1, Table 1). These results indicated that the MCPs may be valid chromosome markers for sorghum chromosome identification.

### 2.2. Chromosome Painting Using MCPs in Sorghum

**Figure 2:**
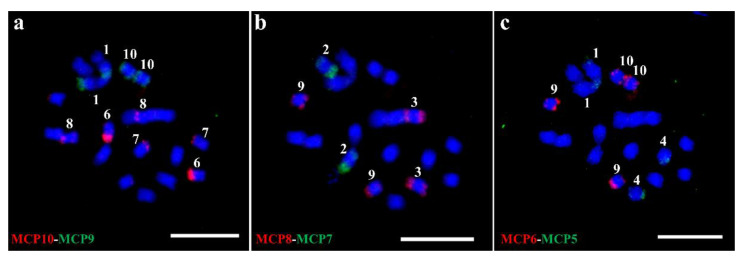
Sequential FISH identifying each of the ten chromosomes in sorghum. (**a**) First round of FISH using MCP10 (digoxigenin-red) and MCP9 (biotin-green) probes. (**b**) Second round of FISH using MCP8 (digoxigenin-red) and MCP7 (biotin-green) probes. (**c**) Third round of FISH using MCP6 (digoxigenin-red) and MCP5 (biotin-green) probes. Arabic numerals denote the chromosome number in sorghum. Bar = 10 μm.

Based on the distributions of the ten MCPs above, we selected six probes, MCP5–MCP10, that were sufficient to distinguish ten sorghum chromosomes in a few rounds of oligo-FISH. These six MCPs were labeled by digoxigenin or biotin, and conjugated with anti-dig or anti-bio antibodies, respectively. Pairs of probes were sequentially hybridized to the same metaphase chromosomes prepared from the root tips of sorghum. For example, MCP9 and MCP10 probes were hybridized to the metaphase cell (Figure 2a). The slide was then washed and re-probed with MCP7 and MCP8 probes (Figure 2b). Finally, all six MCPs probes were applied after three sequential FISH experiments (Figure 2a–c).

Expectedly, our FISH results suggested that the MCPs’ signal patterns were well correlated with the sequence alignment distribution between MCP sequences and sorghum genome (Figure 1 and Figure 2). For example, MCP10 produced variously distinct signals on chromosomes 6, 7, and 8, which presented unique signal types for each of these chromosomes (Figure 2a). However, MCP10 did not produce an observed signal on chromosome 9 (Figure 2a). Furthermore, MCP6 (Figure 1f and Figure 2c) and MCP5 (Figure 1e and Figure 2c) did not produce an observed signal on chromosome 7 and chromosome 10, respectively. These results suggested that the unobservable signal of these probes may be caused by the limited oligo numbers on the chromosomes (Figure S1). In addition, MCP5 did not produce an expected strong signal on chromosome 4 (Figure 2c), which implied that the sequential FISH may affect the efficiency of hybridization.

### 2.3. Chromosome Identification in T. arundinaceum Using MCPs, 5S rDNA, and 35S rDNA Probes

**Figure 3:**
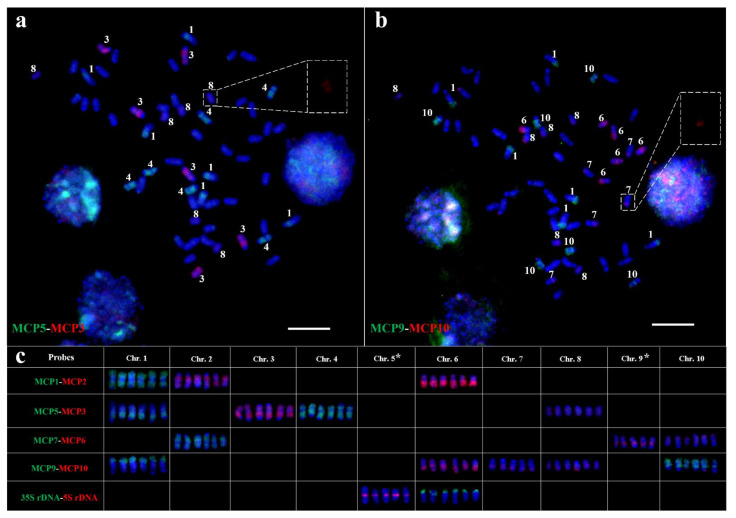
Sequential oligo-FISH identifying all 10 chromosomes on the same metaphase plate of *T. arundinaceum*. (**a**) Second round of oligo-FISH using MCP5 (biotin-green) and MCP3 (digoxigenin-red) probes. (**b**) Fourth round of oligo-FISH using MCP9 (biotin-green) and MCP10 (digoxigenin-red) probes. (**c**) Statistical results of different probes. Arabic numerals denote the chromosome numbers. The dotted box denotes the weak signals of chromosomes 8 and 7. The symbol * indicates chromosomes 5 and 9 were both missing one copy in this particular cell due to the preparation of the slides. The six copies of chromosomes 5 and 9 on metaphase plate are provided in Figure S3. Bar = 10 μm.

So far, the *T. arundinaceum* genome data are still unavailable. It is difficult to establish the karyotype using repetitive sequences or BAC clones. Hence, we attempted to use the same metaphase cell for chromosome identification in *T. arundinaceum*. The sequential FISH prevented cross-hybridization signals from non-target chromosomes. We selected a putative hexaploid *T. arundinaceum* (Hainan92-77, 2n = 60, *x* = 10) for chromosome painting analysis using MCPs. We performed five rounds of sequential FISH using MCPs on the same metaphase plate of *T. arundinaceum*. Based on the distribution of MCP on the sorghum genome, we named the chromosomes 1–10 of *T. arundinaceum* according to the sequential FISH results (Figure 3 and Figure S2). For example, MCP2 and MCP1 were labeled with digoxigenin (red signal) or biotin (green signal), respectively (Figure S2a and Figure 3c). FISH showed that MCP2 produced differential signals on chromosomes 2, 5. and 6 of *T. arundinaceum* (Figure S2a). We named them as chromosomes 2, 5 and 6 of *T. arundinaceum* according to the sorghum chromosome nomenclature (Figure 1b). The MCP1 probe produced distinct signals on six chromosomes of *T. arundinaceum* and we identified them as chromosome 1 in *T. arundinaceum* (Figure 3c). Altogether, all the *T. arundinaceum* chromosomes were identified using the eight MCPs, although the signals varied on different chromosomes. Additionally, the above FISH results between sorghum and *T. arundinaceum* showed that the synteny of all ten chromosomes was conserved over more than 9 MYs of divergence among these two species [17].

We then performed FISH using 5S and 35S rDNAs probes in the fifth rounds of the sequential FISH after MCP. FISH results suggested that 5S and 35S rDNAs sites were located on chromosome 5 and chromosome 6 (Figure S2c), respectively. However, 5S rDNA was close to the centromeric region and 35S rDNA was mapped on the distal region.

### 2.4. Standard Karyotype Analysis of T. arundinaceum Based on Sequential Oligo-FISH

**Figure 4:**
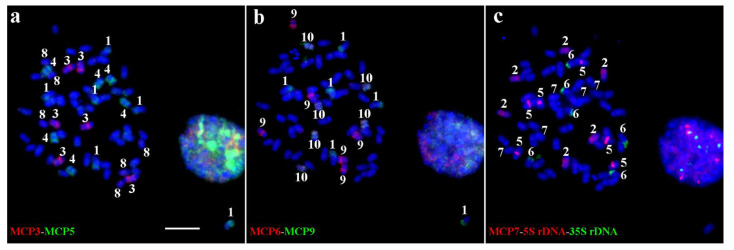
Sequential oligo-FISH identifying all 10 chromosomes on the same metaphase plate of *T. arundinaceum*. (**a**) First round of FISH using MCP3 (digoxigenin-red) and MCP5 (biotin-green) probes. (**b**) Second round of FISH using MCP6 (digoxigenin-red) and MCP9 (biotin-green) probes. (**c**) Third round of FISH using MCP7 (digoxigenin-red), 5S rDNA (digoxigenin-red), and 35S rDNA (biotin-green) probes. Arabic numerals denote the chromosome numbers. Bar = 10 μm.

In order to identify the *T. arundinaceum* chromosomes quickly, we selected seven probes, namely MCP3, MCP5, MCP6, MCP7, MCP9, 5S rDNA, and 35S rDNA. The combined probes could be used to identify *T. arundinaceum* chromosomes with three rounds of sequential FISH (Figure 4). For example, we used MCP3 (red) and MCP5 (green) probes to identify chromosomes 1, 3, 4, and 8 (Figure 4a). Among them, chromosome 8 produced a weak signal. Then MCP6 (red) and MCP9 (green) were selected to identify chromosomes 1, 9, and 10 (Figure 4b). MCP7 (red), 5S rDNA (red), and 35S rDNA (green) probes were used to classify chromosomes 2, 5, and 6 (Figure 4c). Finally, chromosome 7 was classified by excluding nine nonhomologous chromosomes already identified above.

**Figure 5:**
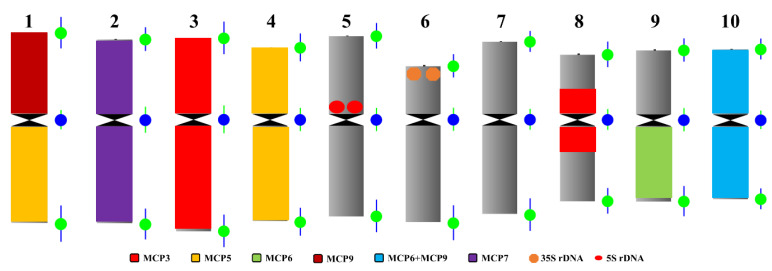
Idiogram of the karyotype in *T. arundinaceum.* The different colors denote the different probes or combined probes.

Identification of chromosomes on the same metaphase plate provided us a standard karyotype based on the individually identified chromosome. After identification of chromosomes 1–10, the centromere probe was also located on *T. arundinaceum* chromosomes for further chromosome measurement (Figure S4). We measured each of ten chromosomes on the metaphase cells for *T. arundinaceum*, then the karyotype was established accordingly (Table 2 and Figure 5). According to the traditional chromosome classification [18], most chromosomes of *T. arundinaceum* are metacentric with the arm ratio ranging from 1.14 ± 0.17 to 1.45 ± 0.21 (Table 2). The idiogram of *T. arundinaceum* was also constructed based on the measured data (Figure 5). Chromosome 1 was the longest (3.58 ± 0.19 μm, Table 2 and Figure 5) and chromosome 8 was the shortest one (2.50 ± 0.23 μm, Table 2 and Figure 5).

## 3. Discussion

The development of the FISH method based on oligo probes provided a powerful tool for understanding the structure, organization, and evolution of plants [7]. However, karyotype analysis is still a huge challenge in non-model plants. Although there are various available DNA probes for FISH in plants, such as repetitive sequences [19] and bacterial artificial chromosome (BAC) clones [20], etc., application in related species that diverged a few MYs ago has always shown an unsatisfactory result. For example, BAC probes are not suitable for FISH in some plant species with large complex genomes [21]. Repetitive DNA probes are the FISH probes used widely in plant genome research, however, such probes always show a varied signal among different species as a result of the instability of genome, so that they cannot be used for cytogenetics research [22]. In *T. arundinaceum*, Yu et al. [23] screened many repetitive sequences; unfortunately, these repeats showed a diversity site that cannot be used as a stable marker for chromosome identification. By comparison, recently, the FISH probe based on single copy sequences designed from genome sequences has provided us a more universal and stable marker for cytological study in plants [15,24]. 

Previous researchers have shown that oligo probes can be applied to chromosome identification in related species that diverged ~12 MYs ago, such as in *Cucumis* [24], or even as long ago as ~15 MYs in *Solanum* species [15]. In our study, MCPs were used to identify all sorghum chromosomes successfully, even though sorghum and maize diverged from the common ancestor about 11.9 MYs ago [25]. Braz et al. [26] tested the maize barcode probes in sorghum, however, it did not produce a sufficient number of signals to identify all sorghum chromosomes. In this case, it could be due to the insufficient number of oligos used. Altogether, these results suggest that chromosome painting probes should have greater potential for related species chromosome identification.

*T. arundinaceum* is an important wild resource for sugarcane breeding. As *T. arundinaceum* is a ployploid plant with 2n = 60 chromosomes (basic chromosome number *x* = 10), the genome sequences are still unavailable, which has greatly hindered the development of cytogenetics research. Although many repetitive sequences were obtained in *T. arundinaceum* [27], none of them have been successfully applied to identify individual chromosomes in *T. arundinaceum*. Here we tested the MCPs derived from maize, suggesting an unexpected signal in *T. arundinaceum*. We demonstrated that the MCPs can be used for chromosome identification in *T. arundinaceum*, even though the divergence time between *T. arundinaceum* and maize is approximately 18 MYs [17]. Furthermore, 5S rDNA was located on chromosome 5 of *T. arundinaceum*, which is quite different from sorghum, in which the locus of 5S rDNA was mapped on chromosome 9 [28]. This result suggests an unidentified chromosome rearrangement between sorghum and *T. arundinaceum*, although the synteny of the ten chromosomes has been conserved based on the oligo-FISH patterns.

There are also many reports about chromosome inheritance between sugarcane and *T. arundinaceum* [29]. Notably, Babil et al. found that there were significant positive correlations between *E. arundinaceus* chromosome and agronomic characterization [4]. However, these results are just based on the counted number of *T. arundinaceum* chromosomes. It is necessary for us to explore the exact chromosome inheritance or significant positive correlation according to the individual chromosome identification in *T. arundinaceum*. In this study, we identified all *T. arundinaceum* chromosomes for the first time using MCPs and classified them 1 through 10 according to the sorghum genome data. These MCPs will be powerful tools for further understanding the chromosome inheritance in the hybrids between sugarcane and *T. arundinaceum*. In addition, individual chromosome identification will dramatically accelerate the research about the exact chromosome, which will contribute to trait improvement in sugarcane breeding.

## 4. Materials and Methods

### 4.1. Plant Material and the Preparation of Metaphase Plates

*T. arundinaceum* (Hainan92-77, 2n = 60, *x* = 10) was maintained at Fujian Agriculture and Forestry University and the root tips were collected from healthy plants. The seeds of *Sorghum bicolor* inbred line BTx623 were used to generate roots at room temperature. Then, the root tips were treated and the slides were prepared according to Braz et al.’s protocol [30] with minor adjustments. Treated root tips were washed in water, then the section containing dividing cells was dissected and digested in enzyme mixture (1% pectolyase Y23, 2% pectinase, 2% RS, and 4% cellulase Onozuka R-10) for 4 h at 37 °C. After digestion, the root sections were washed in water and then washed in Carnoy’s fixative two times briefly. The root sections were carefully broken by using a pipette tip. The suspension cells were dropped onto glass slides and another 10 μL acetic acid were dropped onto them when the slide had almost dried.

### 4.2. Sequence Alignment and Analysis

The sequence of maize CPs is available in the published paper [14] (https://www.pnas.org/content/suppl/2019/01/15/1813957116.DCSupplemental, accessed on 17 January 2019). The MCP sequences evenly cover the entire chromosome sequence of maize with an average oligo density of 0.25 oligo per kb. The sorghum genome was downloaded from the NCBI website (https://www.ncbi.nlm.nih.gov/genome/?term=Sorghum%20bicolor, accessed on 7 April 2017). TBtools software [31] was used to sequence alignment between MCP sequences and the sorghum genome with default parameters. The chromosome location of the ten MCP sequences was drawn by RIdeogram software [32]. We discarded the aligned sequences that were smaller than 32 bp, meaning that at least a 32 bp (70% homology) match with the sorghum genome was required for sequences to be retained and counted.

### 4.3. Oligo-FISH and Karyotype Analysis

5S and 35S rDNAs were labeled with digoxigenin-11-dUTP or biotin-16-dUTP (Roche Diagnostics, Mannheim, Germany) using a Nick Translation Kit (Roche Diagnostics, Mannheim, Germany). The centromere probe was prepared according to Huang et al. [33]. The So1 probe was used to localize the centromeric region, as it has the highest genome proportion and is located on all chromosomes in sugarcane. MCPs were amplified and labeled according to published protocols [30] using a T7 in vitro transcription method. The first round of FISH was performed as described by Braz et al. [30]. All biotin-labeled (~500 ng) probes were detected by anti-biotin fluorescein (Vector Laboratories, Burlingame, CA, USA) and digoxigenin-labeled probes (~400 ng) were detected by antidigoxigenin rhodamine (Roche Diagnostics, Indianapolis, IN, USA). Chromosomes were counterstained with DAPI (4′, 6-diamidino-2-phenylindole). An AxioScope A1 Imager fluorescent microscope (Carl Zeiss, Gottingen, Germany) was used for capturing images. The final image contrast was processed using Adobe Photoshop 21.0.0 software. Measurement of the short arm and long arm of the individual chromosomes was conducted in the DRAWID software [34]. Arm ratio = the long arm/the short arm; 10 metaphase cells were used for measurement on each chromosome.

Slides with high-quality metaphases were retained for sequential FISH. After the first round of FISH and image capture, the slides were washed three times in 4×SSC (10 min each). The slides were then washed three times in 2×SSC at room temperature (5 min each). Finally, the slides were continuously dehydrated in 70% and 100% ethanol series (room temperature, 3 min each), denatured again in 70% formamide at 70 °C for 2 min, dehydrated in a second ethanol series (pre-cooled at −20 °C, 5 min each) and further hybridized with different probes.

## 5. Conclusions

In this study, the MCPs were applied in sorghum and *T. arundinaceum* using stable oligo-FISH. Our results suggest that MCPs can be used as reliable markers for chromosome identification in *T. arundinaceum*. Using this system, for the first time, we were able to identify all chromosomes of *T. arundinaceum* though chromosomes 7 and 8 had a weak signal. The tested MCPs may be a useful FISH marker for further cytogenetics research in the hybrids between *T. arundinaceum* and sugarcane, since genomic DNA probes have been used to distinguish these species’ chromosomes separately.

## Figures and Tables

**Table 1:** The number of maize CP sequences aligned to each of the sorghum chromosomes.

No. of Oligo	Sorghum1	Sorghum2	Sorghum3	Sorghum4	Sorghum5	Sorghum6	Sorghum7	Sorghum8	Sorghum9	Sorghum10
MCP1	27,169	746	892	820	657	689	4812	2772	700	791
MCP2	701	9016	564	521	2613	11,784	405	446	525	524
MCP3	711	546	20,574	550	495	415	463	3761	435	513
MCP4	836	531	600	9900	5120	449	4999	564	484	543
MCP5	7236	539	552	15,991	382	468	411	490	530	2548
MCP6	625	416	507	404	424	322	1390	659	10,918	6910
MCP7	488	17,672	421	422	316	316	387	347	367	415
MCP8	466	402	11,297	420	294	282	313	372	5725	419
MCP9	8544	434	412	411	277	304	296	300	329	9614
MCP10	648	471	501	479	466	9307	2888	3983	1136	481

**Table 2:** Chromosome length and arm ratio of mitotic metaphase chromosomes of *T. arundinaceum*.

Chromosome Number	Long Arm (μm)	Short Arm (μm)	Chromosome Length (μm)	Arm Ratio
Chr.1	2.02 (±0.19)	1.56 (±0.10)	3.58 (±0.19)	1.30 (±0.18)
Chr.2	2.03 (±0.06)	1.54 (±0.10)	3.57 (±0.11)	1.32 (±0.11)
Chr.3	2.08 (±0.21)	1.49 (±0.21)	3.57 (±0.30)	1.42 (±0.24)
Chr.4	1.84 (±0.16)	1.29 (±0.15)	3.13 (±0.22)	1.45 (±0.21)
Chr.5	1.76 (±0.21)	1.49 (±0.13)	3.25 (±0.26)	1.19 (±0.17)
Chr.6	1.80 (±0.90)	0.90 (±0.10)	2.69 (±0.22)	2.03 (±0.37)
Chr.7	1.70 (±0.19)	1.39 (±0.09)	3.10 (±0.24)	1.22 (±0.12)
Chr.8	1.40 (±0.13)	1.10 (±0.14)	2.50 (±0.23)	1.29 (±0.15)
Chr.9	1.42 (±0.16)	1.19 (±0.11)	2.61 (±0.20)	1.20 (±0.18)
Chr.10	1.38 (±0.08)	1.21 (±0.09)	2.59 (±0.12)	1.14 (±0.10)

## Data Availability

Sorghum genome originated from the National Center for Biotechnology Information (NCBI, https://www.ncbi.nlm.nih.gov/genome/?term=Sorghum%20bicolor, accessed on 7 April 2017) database.

## Supplementary Materials

The following are available online at https://www.mdpi.com/article/10.3390/ijms22168539/s1.
