# Supplementary material for: Chromosomal Characterization of Tripidium arundinaceum Revealed by Oligo-FISH
Source: Int J Mol Sci. 2021 Aug 9;22(16):8539. doi: 10.3390/ijms22168539 (PMC8395171; doi:10.3390/ijms22168539)
Supplement: Supplementary file 1 [file ijms-22-08539-s001.zip › ijms-1305931-supplementary.pdf]

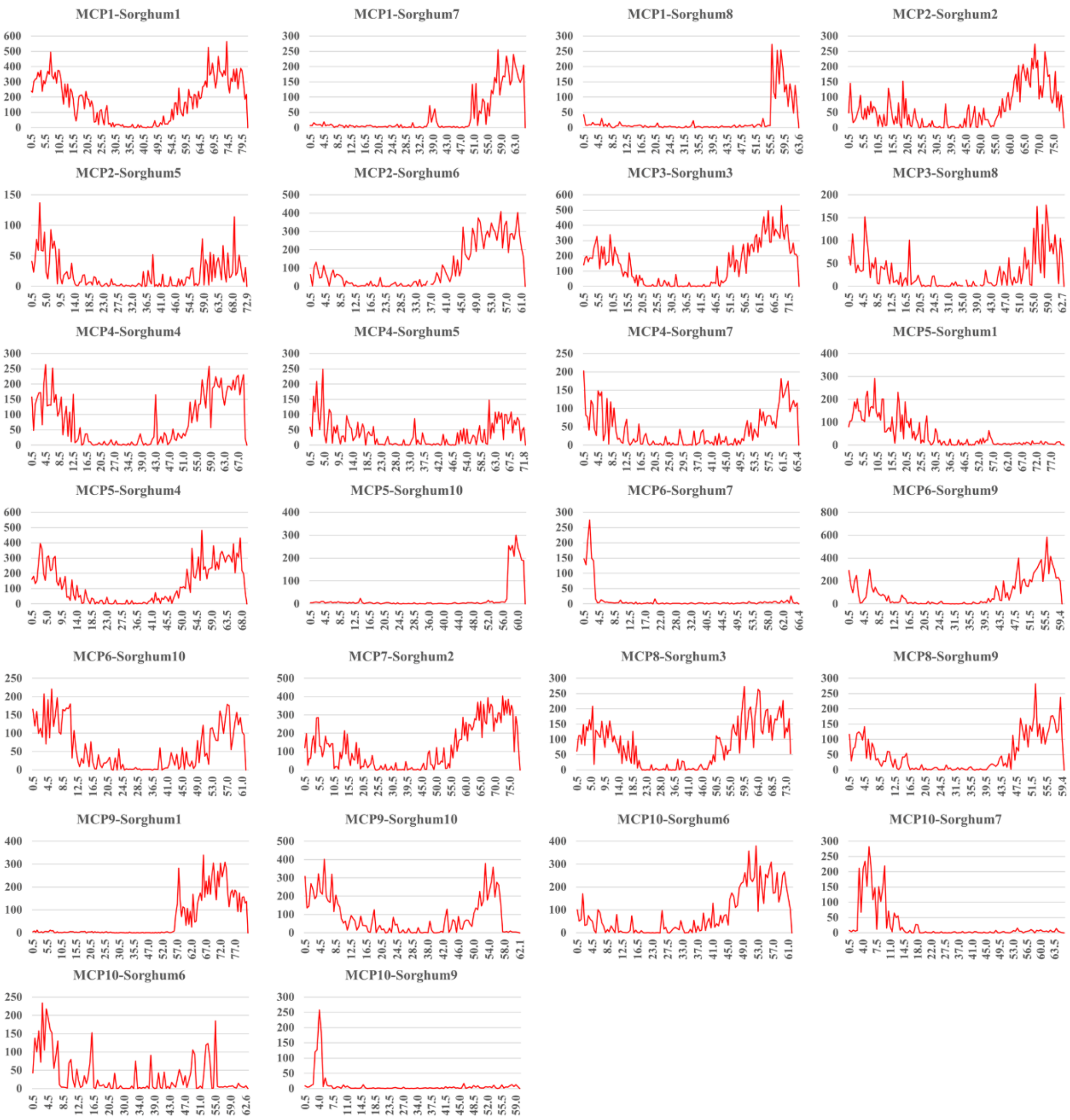

Figure S1. Sequence alignment between MCPs and sorghum genome. The title indicates the MCP1-10 sequences distribution on sorghum chromosomes, respectively. x-axis is the position on the chromosome. y-axis is the number of oligo in each 500-kb window on the chromosome.

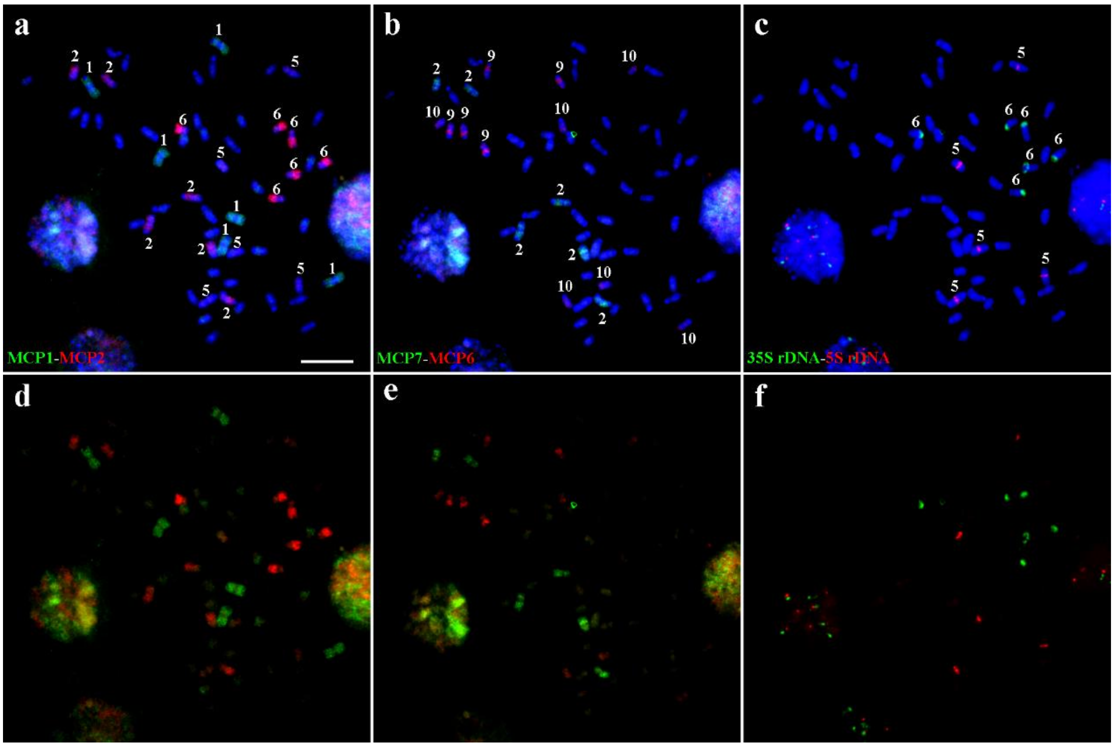

Figure S2. Sequential FISH for chromosome identification in *T. arundinaceum*. (a) First round of FISH using MCP1 (biotin-green) and MCP2 (digoxigenin-red) probes. (b) Third round of FISH using MCP7 (biotin-green) and MCP6 (digoxigenin-red) probes. (c) Fifth round of FISH using 35S rDNA (biotin-green) and 5S rDNA (digoxigenin-red) probes. (d-f) Red and green fluorescence signals were digitally separated from (a-c). Arabic numerals denote the chromosome numbers. Bar = 10 μm.

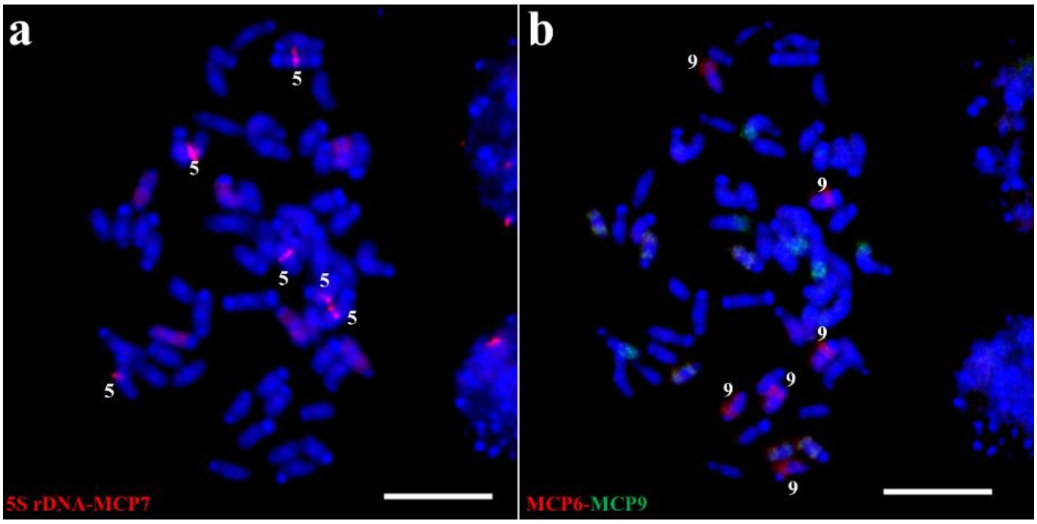

Figure S3. Chromosome identification of *T. arundinaceum* using oligo-FISH. (a) 5S rDNA (digoxigenin-red) and MCP7 (digoxigenin-red) probes. (b) MCP6 (digoxigenin-red) and MCP9 (biotin-green) probes. Arabic numerals denote the chromosome numbers. Bar = 10 μm.

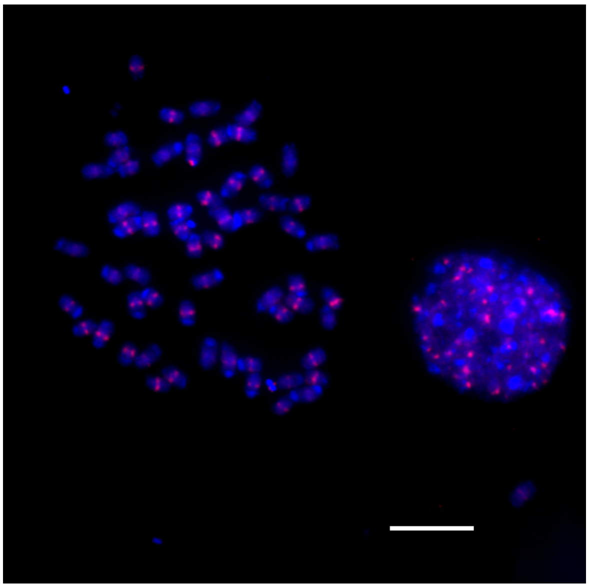

Figure S4. The centromere probe localization by FISH in *T. arundinaceum*. Bar = 10 μm.
